# Milk and Infantile Mortality

**Published:** 1899-02

**Authors:** 


### Milk and Infantile Mortality.

A paper by Dr. Lemiere, which is quoted in the *Lancet*, discusses the relation long since recognized as existing between milk supply and infant mortality. The statistics given are chiefly those of the city of Lille. Among other points of interest noted by the writer is the fact that the death rate under two years of age in that city is 398.6, and in one parish it has risen to 520 for every 1,000 deaths recorded. A large proportion of these is attributed to gastro-enteritis, which is most prevalent in districts inhabited by the working classes, the comparative mortality between a rich and a poor district selected for illustration being as 1 to 7. Gastro-enteritis is, as a rule, a preventable disease, and this fact is fully admitted in the article to which we have referred. It is satisfactory to find that its explanation is sought for where it is most likely to be found—namely, in the diet administered. With regard to this, Dr. Lemiere makes two practical observations—one in which he accuses the employment of watered milk as a cause of malnutrition, and the other in which he advocates efficient sterilization and general are in feeding. Too much importance can hardly be attachedc to these remarks. It is true that they teach nothing new, but they remind us of precautions which should never be but constantly are forgotten. We do not dispute the influence of subsoil water as a factor in the production of infantile diarrhea, but we recognize as of equal and even greater importance the quality of the infants' milk supply. The former consideration is in a great measure *ultra vires*, the later is at all times within our control. If this control is applied with judgment and vigor the climatic and subterranean evils may often be entirely counteracted. Nothing, therefore, in domestic management is more important than care in the selection of the milk used and in its after-treatment. The health of the cattle producing it, of the people who sell it, and the cleanliness of persons and vessels connected with its sale are as much the concern of a nurse or mother as is its actual preparation for the infant. As a rule, mothers in this country understand the importance of cleanliness in all the manipulations of artificial feeding. Nevertheless they are frequently in error. They are very apt to underestimate the value of fresh air, especially at night, and many an infant is poisoned with milk which has fermented in a close bedroom. We need hardly impress upon medical practitioners the necessity of instructing their patients in this matter and also with regard to the means adapted for the ready sterilization not only of milk, but also of the vessels in which it is contained and administered. —*Maryland Med. Journal.*

